# Rigor & Reproducibility: pH Adjustments of Papain with L-Cysteine Dissociation Solutions and Cell Media Using Phenol Red Spectrophotometry

**DOI:** 10.3390/bios15110727

**Published:** 2025-11-01

**Authors:** Joshua M. Hilner, Allison Turner, Calissa Vollmar-Zygarlenski, Larry J. Millet

**Affiliations:** 1 Department of Mechanical, Aerospace, and Biomedical Engineering, The University of Tennessee-Knoxville, Knoxville, TN 37996, USA; 2 Biology Department, Texas State University, San Marcos, TX 78666, USA; 3 Department of Civil and Environmental Engineering, The University of Tennessee-Knoxville, Knoxville, TN 37996, USA

**Keywords:** phenol red, pH indicator, papain, enzymatic digestion, neuron culture, scientific rigor, reproducibility, EDTA, L-cysteine

## Abstract

Phenol red is a widely used, low-cost, label-free colorimetric pH indicator that bridges traditional colorimetric assays with modern quantitative imaging and cell-based screening platforms. Its protonation-dependent absorbance shift (430–560 nm) allows for the real-time monitoring of extracellular acidification, which indirectly reflects cellular metabolism, growth, and respiration. Although phenol red lacks the molecular specificity of genetically encoded or fluorogenic biosensors, it remains useful in systems where pH changes are effective proxies for physiological processes. Existing tissue digestion protocols often overlook key parameters, especially pH control and enzyme cofactor use. This study presents a straightforward, spectrophotometric method to monitor and adjust the pH of low-volume (1 mL) buffered enzymatic dissociation media using phenol red and a plate reader. We titrated dissociation solutions to physiological pH (~7.4) using spectrophotometric pH measurements validated against conventional glass pH probe readings, confirming method reliability. Accurate pH assessment is critical for isolating viable primary cells for downstream applications such as tissue engineering, single-cell omics, and neurophysiological assays. We highlight that papain-based dissociation media supplemented with L-cysteine can be acidic (pH 6.6) if unadjusted, compromising cell viability. This accessible approach enhances reproducibility by promoting pH documentation concerning dissociation conditions that contribute to advancing consistency in biomedical, cellular, neuronal, and tissue engineering research.

## 1. Introduction

Enzymatic digestion is widely employed to dissociate tissue samples by breaking down the extracellular matrix (ECM), thereby enabling downstream applications such as cell culture and manipulation, tissue engineering, and molecular analyses [1,2,3,4,5,6,7,8,9,10,11,12,13,14,15,16,17,18,19,20,21,22,23,24,25,26,27,28,29,30,31,32,33,34,35,36,37,38,39,40,41,42,43,44,45,46,47,48,49,50]. Achieving consistent dissociation of tissues into viable single-cell suspensions requires rigor and reproducibility, as the efficiency of this process directly affects both the quality and quantity of cell recovery, as well as long-term reproducibility and biological accuracy. Despite its foundational role in experimental workflows, a lack of standardized reporting practices in dissociation protocols has introduced significant inter-laboratory variability that could undermine the translational relevance of in vitro model systems designed to emulate in vivo environments. Ambiguity and ad hoc protocol modifications pose challenges for the development of organ-specific tissue models, where even minor deviations in protocol, such as enzyme concentration, temperature, or pH, can alter cell populations, and the structural and functional fidelity of the recovered cells. [Note: in the context of RNA sequencing, regenerative medicine, and mechanistic cellular studies, optimal cell viability and minimal procedural stress are crucial to preserving physiological gene expression and minimizing technical noise]. Thus, every phase of tissue dissociation, from harvesting and handling to enzymatic digestion, exerts influence on the sample’s integrity and downstream analytical outcomes [51].

Efforts to standardize and benchmark cell isolation methods are gaining traction, especially for high-resolution applications such as single-cell transcriptomics, which demand uncompromised sample quality [52,53,54,55]. Table 1 consolidates the best current practices and mechanistic insights into critical dissociation parameters that impact the success and reproducibility of neuron cell isolation.

Our analysis of the literature identified gaps in pH reporting for tissue digestion protocols, particularly regarding neuronal cell harvest. Despite the widespread use of enzymes for tissue dissociation, the critical role of pH is often overlooked. To address this, we leverage phenol red, a common pH indicator in balanced salt solutions, to monitor pH changes during enzymatic digestion. When coupled with optical detection, such as plate readers, phenol red acts as a chemo-optical sensor. Its chemical response to hydrogen ions (H^+^) enables optical measurement of light absorbance to quantify pH. In our application, we use this method with optical plate readers to enable real-time assessment of reaction conditions during the enzymatic digestion of cellular connections. This approach demonstrates how a traditional colorimetric indicator can be integrated into modern high-throughput platforms, showcasing phenol red’s continued utility in contemporary analytical methods.

In this study, we used three media varieties, Dulbecco’s Modified Eagle Medium DMEM (bicarbonate–CO_2_), Hibernate-A (HEPES/bicarbonate), and TrypLE Express (HEPES/phosphate), each with different buffering systems to provide stable pH conditions (7.2–7.4). The stable pH is critical for preserving enzyme activity and tissue integrity during dissociation by maintaining optimal ionization of catalytic residues and extracellular matrix components. The color indicator phenol red was used to monitor the media’s pH, with a shift toward red/purple indicating alkalinity and a shift toward yellow indicating acidity.

In our lab, we switched to a papain enzyme preparation that includes L-cysteine to enhance cell recovery from older tissues. We found that L-cysteine, an activating cofactor, caused significant cytotoxicity when harvesting primary neurons. This toxicity was due to the acidification of the dissociation medium. We found that adjusting the pH of the enzyme solution to a physiological range completely restored cell viability. Our study illustrates the importance of documentation and careful attention to dissociation conditions. We review the existing scientific literature on tissue digestion, highlight the critical role of pH, and focus specifically on the use of papain and its cofactor L-cysteine. The methods and data presented offer a practical approach for titrating enzyme and media solutions for physiological tissue digestion, a process that could be broadly beneficial in cell biology and tissue engineering.

## 2. Materials and Methods

Overview: Calculated and measured pH values for papain-containing solutions, TrypLE Express, and a few standard media formulations are measured using a conventional small probe pH meter; in parallel, we also calculate pH using phenol red as a pH indicator in spectrophotometry. pH measurements and calculations were cross validated by a separate lab technician who was unfamiliar with pH measurement or calculation. Three media were titrated with distinctly different buffers, each were sampled in triplicate through independent replicates, Gibco DMEM/F-12 (A4192001), TrypLE Express Enzyme (12605028), Hibernate-A Medium (A1247501)). Papain enzyme containing L-cysteine and EDTA (Worthington Biochemical, Lakewood, NJ, USA, PAP2, Cat#: LK003178) was used in Hibernate-A Medium. The sample pH was measured using a low-volume pH probe (Thermo, Waltham, MA, USA (Orion), 9110DJWP) connected to a pH meter (ALT (Denver Instrument, Castle Rock, CA, USA) 20552). The absorbance of the samples was measured across a 300–800 nm spectrum using a Varioskan LUX Multimode Microplate Reader (Thermo) and the sample pH values were determined from the resulting absorbance data. Enhancing the rigor and reproducibility of this process should improve the quality of primary cell dissociation for the scientific community.

pH Measurements: The pH meter remained in a storage solution (Thermo, 910001) and underwent calibration with stock solutions of pH 4, 7, and 10 (Thermo, 910104, 910107, 910110). After each measurement, the probe was rinsed with distilled (DI) water and blotted with a Kimwipe to preserve sample fidelity. The values were recorded following measurement stabilization, as indicated by the pH meter. For the papain solution, a 100-unit vial of lyophilized papain manufactured with L-cysteine (1 mM) and EDTA (0.5 mM) was brought to room temperature and reconstituted with 5 mL Hibernate-A medium. The pH of Hibernate-A, a HEPES buffered media, was recorded before and after papain addition. Sample aliquots (1 mL) were titrated using NaOH (1 M and 5 M) and HCl (37%, 12 M) solutions in a polypropylene 96 deep-well (2.2 mL) plate to obtain pH step values for DMEM/F-12, TrypLE Express, and for Hibernate-A with and without papain enzyme. Additional sample aliquots were titrated to surpass the indication range of phenol red (6.8–8.2, Phenolsulfonphthalein CID 4766) [56] for use in absorbance calculations. The samples were thoroughly mixed, and 250 µL aliquots were placed in a clear-bottom 96-well plate for absorbance spectroscopy of phenol red. Well plates contained the titrated samples across pH step values, including acidic (pH < 6.8) and basic samples (pH > 8.2), as well as a sample of stock media.

pH Calculations: Absorbance values of the 250 μL aliquots were measured across a spectrum of 300–800 nm. The absorbance values corresponding to each pH sample were averaged prior to calculating the pH. The absorbance spectrum of phenol red features two distinct peaks, one around 430 nm and another near 560 nm, with their intensities reflecting the solution’s acidity or basicity. A dominant peak at 430 nm indicates an acidic environment, whereas a stronger peak at 560 nm suggests alkalinity [57]. These peaks converge at an isosbestic point around 480 nm, where absorbance remains constant regardless of pH. Once the indicator reaches either extreme (fully protonated below pH 6.8 or fully deprotonated above pH 8.2) the spectral maximum remains stabilized. The absorbance ratio provides a concentration-independent assessment of sample pH using absorbance values (Equation (1)).

(1) $$R=\frac{560\;nm}{430\;nm}$$

The *pKa* of sample solutions was calculated using a modified version of the Henderson–Hasselbalch equation [58] that utilizes the stock pH of the solution provided by the manufacturer and the absorbance values of the stock solution and sample solutions that surpass the indicator range of phenol red (Equation (2)). The absorbance values of the stock solution are described by *A*_430_ and *A*_560_ respectively. The absorbance of the acidic sample (pH < 6.8) at 430 nm and the absorbance of the basic sample (pH > 8.2) at 560 nm are, respectively, described by *A_acid_* and *A_base_*.

(2) $$pKa=pH_{stock}-log(\frac{A_{430}-A_{acid}}{A_{base}-A_{560}})$$

The pH of sample solutions is calculated from the determinations of Equations (1) and (2) (Equation (3)) [59]. The *R_min_* and *R_max_* are determined from the acidic and basic samples, respectively. The value for R is equivalent to the ratio from Equation (1) for the desired pH step value.

(3) $$pH=pKa+log(\frac{R-R_{min}}{R_{max}-R})$$

Hippocampal neuron cultures: Primary hippocampal neurons were isolated from postnatal day 1 to 3 Wistar Kyoto rats following protocols approved by the University of Tennessee Institutional Animal Care and Use Committee, which adhered to all relevant state and federal regulations. Bilateral hippocampi were dissected in cold Hibernate-A (Brain Bits, Springfield, IL, USA) on ice, incubated at 37 °C for 30 min in Hibernate-A containing 20.0 U/mL papain (Worthington Biochemical Corp., Papain PAP2 (Cat#: LK003176) with 1.5 µL NaOH (5 M) in continuous rolling agitation (rotisserie with 5 cm radius, 8.0 RPM). Post digestion, tissues were rinsed with enzyme-free Hibernate-A and mechanically dissociated using fire-polished Pasteur pipettes in 2 mL of supplemented Hibernate-A. The resulting cell suspension was allowed to settle, and the supernatant, containing the dissociated cells, was collected. This gentle 2 mL trituration process was repeated once more to maximize yield. Cells were then centrifuged at 1400 rpm for 5 min, resuspended, counted, and diluted in Neurobasal-A medium (Invitrogen, Waltham, MA, USA) supplemented with 0.5 mM L-glutamine, Gem21 NeuroPlex (Gemini Bio-Products, West Sacremento, CA, USA), 100 U/mL penicillin, and 0.1 mg/mL streptomycin. Cells were plated at a density of 100–125 cells/mm^2^ in poly-D-lysine coated 35 mm Petri dishes and maintained in vitro for 7 to 21 days.

Generative AI: GenAI (ChatGPT-5) was used to generate foundational aspects of the graphical abstract. GenAI was not used for study design, data collection, analysis or interpretation.

## 3. Results

**Figure 1:**
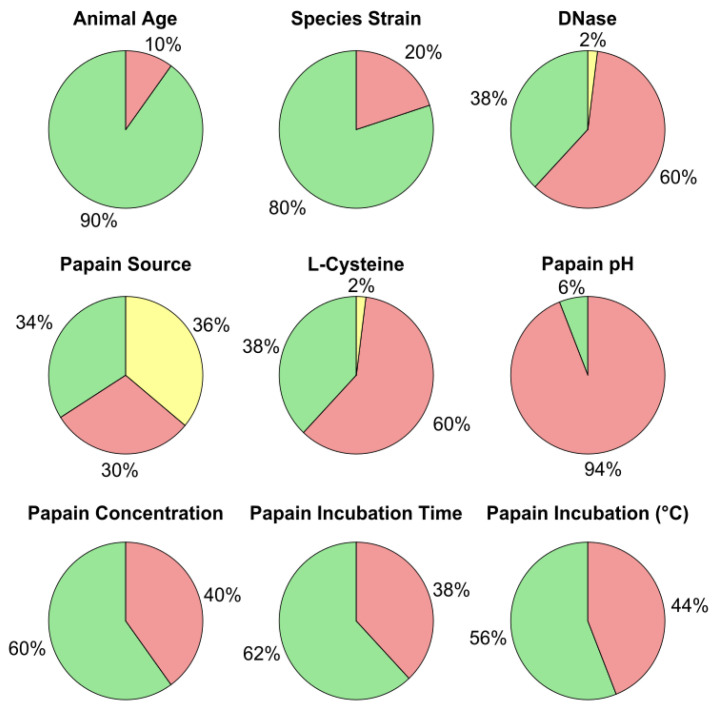
Proportions of relevant experimental details reported in the literature sample. Green shading represents a defined value in the report, red indicates an absence, and yellow addresses the parameter, but the details are not defined.

A literature search sampling the recent 15-year period (2009–2024) framed a better understanding of reporting parameters. Search terms, “papain AND primary AND neuron”, and “papain AND primary neuron culture” for three databases: PubMed, Scopus, and Science Citation Index Expanded. The search returned over 17,000 results. Publications used to assess reported parameters were defined as those with more than 100 citations. Of the resulting list, 40 publications had 100 or more citations, and 10 publications had 20 or more citations. Relevant tissue processing criteria are summarized in Table 2, including parameters such as the animal tissue source, brain region, age, papain concentration (mg/mL), digestion media formulation (Media), units of activity used (U.), incubation time (min) and temperature (°C), manufacturer and catalog number, pH, enzyme cofactor (if any), and secondary enzyme (DNase). A summary analysis highlights a lack of reporting on procedures and conditions among the sampled literature (Figure 1), particularly the absence of papain source, L-cysteine cofactor, pH, concentration, time, and temperature.

### 3.1. Analysis of Published Protocols

Figure 1 summarizes nine parameters used in tissue dissociation for cell culture, as identified from the literature survey. Key inconsistencies in reporting these parameters are also highlighted in Figure 1. Animal age and species/strain were reported in 80% to 90% of the results. Deoxyribonuclease (DNase), a supplementary enzyme, was explicitly reported in 38% of protocols, broken links or aged out description related to changes in manufacturer account for the 2% not available results (N/A). The papain source and product number appear to be listed in 34% of the publications, while an additional 36% (yellow) provide some product details without manufacturer name, and 30% do not report papain source or product information. The papain cofactor, L-cysteine, was supplemented in 38% of the studies. Papain pH is mostly not reported; this occurs in about 94% of the publications. The papain digestion values frequently reported are the concentration (listed in 60% of reports), incubation time (detailed in 62% of studies), and incubation temperature (reported in 56% of studies). Of the 60% of protocols that report the concentration of papain used, one-third provide concentration details in mg/mL instead of ‘units of activity’ or ‘activity units.’ This percentage is not represented in the papain concentration pie chart. Reporting concentration in U/mg or U/mL provides a standardized measure of enzyme activity and potency, which can be used to calculate digestion units (activity units × time). None of the 50 manuscripts sampled mention all of the parameters displayed in Figure 1, which are determined to be key factors for preserving rigor and reproducibility in neuron dissociation using the papain enzyme. Aside from pH consideration as a reporting variable, there are at best three publications that meet all other parameters for reporting. More specifically, two additional publications report all variables, excluding pH, but do not involve papain’s cofactor, L-cysteine, in the dissociation process. One publication reports all parameters except for the strain of rat used in the experimental processes, in addition to not mentioning pH.

**Figure 2:**
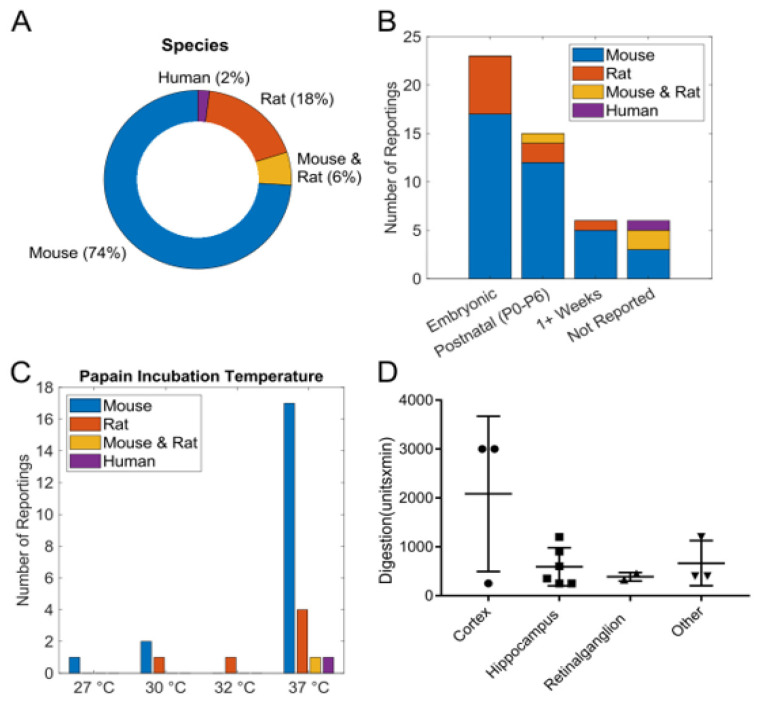
Key findings from the literature reveal the species and age of tissue for neuronal extraction, the incubation temperature of papain, the brain region of origin for neuronal cells, and the digestion units. (**A**) The order of prevalence for reported experimental species is mouse, rat, a combination of mouse and rat, and human. (**B**) Neuronal extraction occurred predominantly in the embryonic stage in most reports, but extraction is also performed from P0–P6 and is less prevalent past one week. (**C**) The reported incubation temperature for papain digestion ranges from 27 °C to 37 °C with the highest neuronal cell population at 37 °C. (**D**) The cortex, hippocampus, and retinal ganglion regions were the primary sources of reported neuronal cells. Digestion units are the mathematical product of enzymatic activity units and incubation time in minutes, reaching a maximum of nearly 4000 digestion units.

Figure 2 summarizes indications of tissue source and digestion activity specific to the source animal tissue. Figure 2A shows that most studies (74%) report mouse as the source tissue, followed by rat (18%), both mouse and rat (6%), and occasionally human (2%). In over 80% of reports where age could be identified, the vast majority specified embryonic or early postnatal tissue as the source. Only a few publications reported using tissue from 1-week-old animals, and none mentioned adult tissue. Some studies did not report age at all (Figure 2B). Most studies report papain use at physiological temperature (37 °C), but a few studies use significantly lower temperatures for papain digestion (Figure 2C). Most tissue digestion units (papain units x time), when reported, are independent of tissue type (Figure 2D).

### 3.2. pH Measurements and Calculations

**Figure 3:**
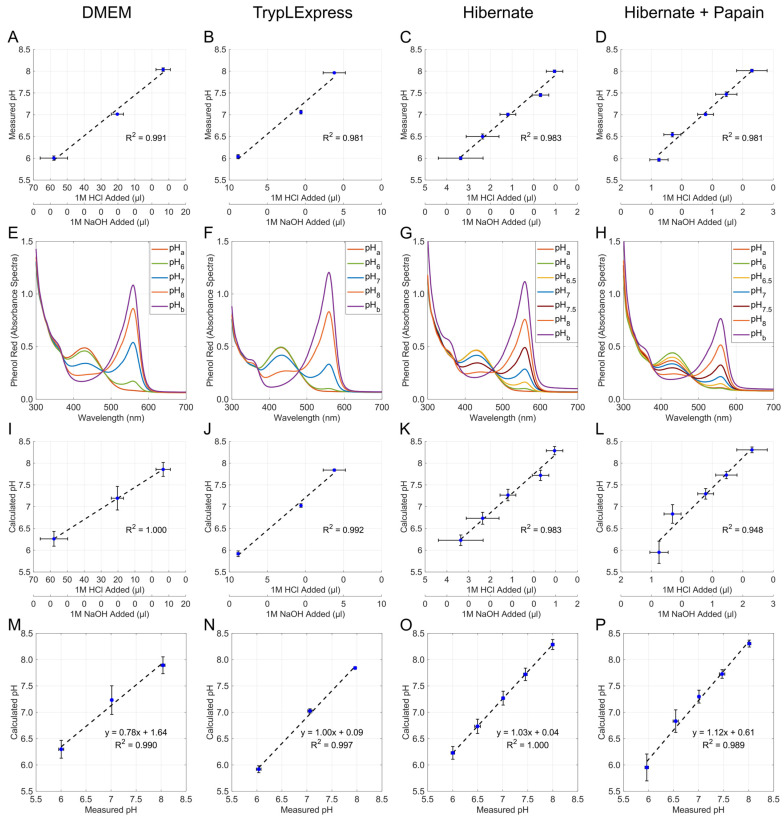
Measured and calculated pH values obtained from glass pH meter measurements and phenol red absorbance calculations for DMEM F/12, TrypLE Express, Hibernate-A, and Hibernate-A + Papain. (**A**–**D**) Sample pH measurement by pH meter for target pH step values relative to titration amounts of 1 M HCl and 1 M NaOH yields R-squared values representative of linear relationships. (**E**–**H**) Absorbance spectrum of phenol red (300–700 nm) produced by media at pH step values exhibiting 430 nm and 560 nm maxima and an isosbestic point at 480 nm. (**I**–**L**) Sample pH determination by absorbance calculations for target pH step values relative to titration amounts of 1 M HCl and 1 M NaOH yields R-squared values representative of linear relationships. (**M**–**P**) Plot of measured versus determined pH values for each media exhibiting linearity marked by R-squared values > 0.98 and accuracy as determined by slope relative to 1.0. (**A**–**D**,**I**–**P**) Blue dots represent the mean with corresponding black error bars (standard deviation).

To demonstrate and aid in improved reporting of tissue digestion parameters, digestion media pH was measured and reported through two methods, a glass microprobe pH meter (Figure 3A–D) and absorbance spectroscopy of phenol red media (Figure 3E–H).

In Hibernate-A media, the reconstitution of papain containing L-cysteine and EDTA yields a significant acidification (pH 6.6), which is detrimental to neurons. Summary values for the volume of acid or base required to shift the media pH to half or whole unit changes are provided below the X-axis. (Figure 3A–D,I–L). The slope and correlation coefficient (R^2^) between measured and calculated pH values are shown (Figure 3M–P) with a strong linear relationship for pH data points.

**Figure 4:**
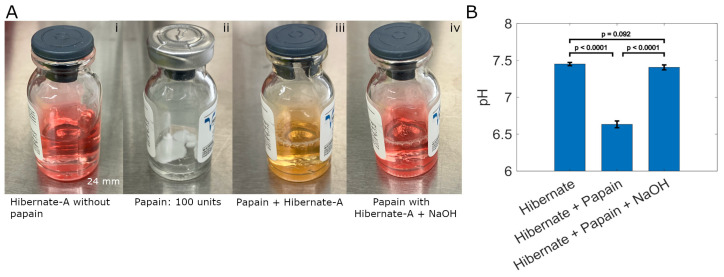
Restoration of Hibernate-A media to physiological pH (7.4). (**A**) (i) Hibernate-A with phenol red pH indicator prior to combining with papain; 5 mL of the same media was added to a glass vial and shown here for visual comparison. (ii) Lyophilized papain (containing L-cysteine and EDTA) at 100 units/vial. (iii) Lyophilized papain vial from (ii) combined with 5 mL Hibernate-A containing phenol red shows significant acidification. (iv) Addition of 5 M NaOH (1.5 µL) restores dissociation solution pH to physiological level (pH 7.4). (**B**) pH measurements of Hibernate media before papain (pH 7.4), containing papain + L-cysteine + EDTA (pH 6.6), and following the addition of 1.28 ± 0.3 µL 5 M NaOH to increase alkalinity that elevates the pH to 7.4 (±0.03).

Figure 4 shows the phenol red status and corresponding pH values in Hibernate-A media formulations used for brain tissue digestion. The sequence of use (i–iv) for papain containing L-cysteine (a papain cofactor) and EDTA (a calcium chelator) in Hibernate-A medium is illustrated in Figure 4A. These components are responsible for an approximate drop of 0.77 pH units from the physiological pH (7.4) of the buffered Hibernate-A media. When analyzed in isolation at the molar ratios present in the papain composition, L-cysteine affects a pH change of ~0.76 and EDTA of ~0.68 units towards acidity. Statistical significance of pH (*p* < 0.0001, two-tailed *t*-test) change is shown between both the stock and final Hibernate-A media when compared to the pH 6.6 ± 0.1 of reconstituted papain (Figure 4B). The pH is restored to ~7.4 with addition of 1.28 ± 0.36 µL of 5 M sodium hydroxide. The difference between the stock Hibernate-A media and final solution pH is not statistically significant (*p* = 0.092, unpaired *t*-test) (Figure 4B) for multiple papain vials.

### 3.3. Culture of Isolated Neurons

**Figure 5:**
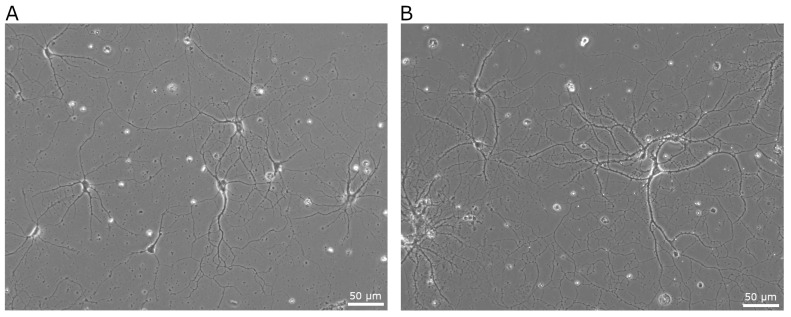
Hippocampal neurons cultured following papain digestion (30 min, pH 7.4) and dissociation from early postnatal rat brain. Micrographs show CA1-CA3 hippocampal neurons grown on poly-D-lysine in vitro for 8 days (**A**) and 42 days (**B**). Hippocampal tissue digested with unadjusted papain (30 min, pH 6.6) resulted in extensive cell death resulting in a debris field.

Successful establishment and maintenance of primary hippocampal neurons was achieved through an unbiased independent effort. Following papain digestion at pH 7.4 for 30 min, hippocampal neurons demonstrated healthy attachment and progressive growth over 42 days. In comparison, digestion with unadjusted papain (pH 6.6) containing L-cysteine and EDTA caused widespread cell death and accumulation of cellular debris. Micrographs of hippocampal neurons maintained in culture for 8 and 42 days are presented in Figure 5.

## 4. Discussion

While phenol red is a standard, qualitative pH indicator in most cell culture media, its potential for quantitative measurement is often overlooked. We addressed this limitation by developing a simple, low-cost colorimetric assay that leverages modern spectroscopic detection via multi-well plate readers. A strong, positive linear relationship was observed between the quantitative pH results and those measured by the conventional method. This quantitative method effectively transforms the familiar colorimetric shifts of phenol red into a useful biosensing tool. This advancement offers a practical solution for a variety of critical biological applications, such as maintaining the precise pH stability required for primary neuron viability.

The light absorption properties of phenol red shift with changes in proton concentration, causing a visible color change. This optical signal serves as a pH indicator in biological media, allowing researchers to indirectly monitor cellular growth, respiration, and metabolic activity through changes in extracellular acidification. Since careful pH monitoring is essential for cell viability and proliferation, phenol red is a valuable tool despite its limitations. With measurable absorbance changes typically between 430 and 560 nm, it offers a simple, if less specific, alternative to advanced biosensors for applications where pH serves as a convenient proxy for physiological processes.

Standardization and transparent reporting of tissue processing conditions are critical for improving the reproducibility and interpretability of experimental findings [60,61,62,63,64,65,66,67,68]. Enzymatic digestion, in particular, is highly sensitive to environmental variables such as pH, temperature, buffer composition, and enzyme concentration. These factors must be carefully controlled and reported, as even subtle deviations can alter digestion efficiency, compromise cell viability, or introduce artifacts into downstream analyses.

Phosphate-buffered saline (PBS) is one of the most commonly used buffers in biological research due to its isotonicity and non-toxic ionic composition. However, while PBS is ideal for washing and short-term handling of cells, it lacks the nutrients, pH stability, and protective additives that support cell function during extended enzymatic digestion. Therefore, PBS should be viewed as a short-term handling buffer rather than a functional digestion medium.

Among the enzymatic media formulations tested, TrypLE Express is most similar to PBS in its CO_2_-independent buffering capacity, achieved through phosphate and HEPES buffers. It is a bench-stable solution specifically formulated for gentle cell and tissue dissociation. We selected TrypLE Express for testing because of its widespread use in dissociating adherent cells, and its formulation offers a gentler, more stable, and animal origin–free alternative to traditional trypsin.

Dulbecco’s Modified Eagle Medium (DMEM) is a widely used cell culture medium that relies on a sodium bicarbonate buffer system and requires a 5–10% CO_2_ atmosphere to maintain physiological pH. Multiple DMEM formulations are available, varying mainly in glucose concentration and additional supplements. In contrast, Hibernate-A is a CO_2_-independent, serum-free basal medium optimized for maintaining neuronal cells and tissues under ambient conditions. It is specifically designed for benchtop procedures such as tissue dissociation, microscopy, flow cytometry, and transport where a CO_2_ source is not available.

We recognize that Table 2 shows more media formulations in the spectrum of buffered solutions (e.g., HBSS, EBSS, PDS, and custom formulations). A range of papain formulations exist (suspension, lyophilized, with or without L-cysteine, EDTA), with different availability and product costs.

Enzyme choice shifts the yield/viability balance in recovering cells [60,61], and can be performed to influence epitope preservation without a loss in viability for scRNA sequencing [62]. To improve cell yields and viability, alternative, combined, or sequential enzymatic approaches may be performed to improve cell recovery for retrieving target cell types, for example, non-antigen-presenting mast cells and CD8 T cells from skin [63]. Conversely, to safeguard against reduced cell viability when releasing cells from tissues, an increased incubation time and enzyme concentration should be evaluated, as it has the potential to reduce cell viability [64,65].

Excessive or insufficient enzymatic digestion, temperature changes, and treatment with or without mechanical assistance influence variability, which could compromise data quality and bias gene expression [66,67]. The inclusion of inhibitors for RNA polymerase and transcriptional activity combined with mechanical dissociation protocols has been shown to prevent artifactual changes in gene expression associated with enzymatic dissociation [68]. Lower temperature digests have been shown to prevent transcriptional heat shock stress [67], but the feasibility of operating at lower temperatures is determined by enzyme sequence and structure.

Enzymatic dissociation protocols typically operate within a recovery range where yield and viability parameters are maximized through optimization, but the influence of the protocols on cellular gene expression needs greater attention.

The careful preparation of clean, healthy cellular suspensions is essential for optimal seeding into nanoliter-scale microfluidic devices and for obtaining reliable results in signaling or live-cell imaging studies [69]. Incomplete or suboptimal digestion, often due to enzyme degradation, can lead to cellular clumping, in which live and dead cells, tissue debris, and extracellular DNA become entangled. DNase is often added to dissociation protocols to prevent DNA-mediated clumping and to maintain a uniform cell suspension. However, instead of relying on DNase to clear cell-free DNA, maintaining and monitoring for adequate enzyme activity is advised. This can be achieved by inspecting the slurry for DNA clumps in each digestion, a simple checkpoint to signal the need for fresh enzyme.

To better understand contributors to pH shift, we considered the effects of the L-cysteine cofactor (1 mM) and EDTA (0.5 mM), both of which are present in the reconstituted lyophilized papain digestion solution. Phenol red is an efficient indicator for rapid determination of acidity and alkalinity [57], and it provides practical, real-time feedback for a quick and low-cost strategy to standardize pH across experiments. If phenol red is omitted from dissociation protocols to avoid potential effects on metabolism or gene expression, the media pH should be tested. Alternatively, L-cysteine could be manually added to other papain products, then separate optimization experiments should be performed to define ideal operational conditions that should be reported in the literature. Given the simplicity of pH measurement, its inclusion as a routinely reported parameter would strengthen science involving tissue dissociation.

Off the shelf kits and mechanical tissue dissociation have the potential to provide greater consistency between experiments, but they may need to be analyzed and adjusted for optimal viability and yield. Understanding how the yield–viability relationship shifts across organ systems, tissue architectures, and cell phenotypes, and how it influences specific experimental applications, offers a path toward improving the rigor and reproducibility of cell-based assays and tissue-derived models. Optimization strategies including cell quantitation and characterization procedures are summarized within the Worthington Biochemical Tissue Dissociation Guide [70].

## 5. Conclusions

Physiological pH is a critical, yet often underreported, factor for cell health during and after primary tissue dissociation. Phenol red spectrophotometry offers a rapid and low-cost method for measuring and defining the pH of dissociation conditions, achievable with many standard plate readers. During enzymatic dissociation, particularly with proteases like papain, tight regulation of pH and the proper use of enzymatic cofactors are critical for preserving cellular viability and maximizing yield. A high number of low-quality cells, often resulting from suboptimal conditions, undermines experimental integrity. Matching tissue mass with enzymatic strength and incubation time promotes productive dissociation and can eliminate the need for DNase to resolve clumping, an outcome that often indicates under-digestion.

With standardized protocols, even novice researchers—such as undergraduates or summer scholars—can generate and maintain healthy neuronal cultures. Such consistency is particularly important in neuroscience, where small variations in dissociation conditions can alter the recovery and gene expression profiles of fragile neuronal populations, ultimately impacting functional and omics-based assays.

## Figures and Tables

**Table 1:** Summary of factors that influence optimization of enzymatic digestion.

Domain	Key Considerations	Research Implications
Enzymatic Variability	Variability in published protocols: enzyme types, storage, activity units, incubation times, and buffer conditions	Underscores the need for harmony and transparency in methods
Minimize Mechanical Stress	Gentle dissociation reduces cellular stress and clumping Preservation of fragile cell types	Improve viability and yields for tissue engineering and single-cell analysis
Tissue-Specific Enzyme Tuning for Neuroanatomical Regions	ECM composition Glial density, fiber tracts, and vascularity Brain or spine tissue source Region-specific dissociation protocols	Enhances cell recovery Reduces batch variation Demands region-specific adjustments to isolate intact, and representative cell types
Avoiding Population Skew	Harsh and weak enzymatic conditions bias robust cell types and deplete fragile populations	Affects conclusions for studies using heterogenous cell populations
Age-Dependent Tissue Maturity	Postnatal tissues: more dense cellular connections Embryonic tissues: more effective enzymatic digestion	Requires stage-matched dissociation Shapes cell survival, phenotypic stability, and experimental relevance
Oxidative Stress	Neurons are highly susceptible to ROS due to high metabolic rates and low antioxidant defenses	Elevated oxidative stress impairs viability and biases downstream functional assays

**Table 2:** Summary results of primary neuron culture parameters from 50 highly cited publications (15 yrs: 2009–2024).

Ref.#	Year	Citations	Source	Region	Age	mg/mL	Media	U.	Min.	°C	Mfr.	Cat. No.	pH	L-Cys.	DNase
[1]	2009	124	Mouse	C	X	x	Custom	100	30	37	S-A	x	yes (7.4)	Use	x
[2]	2019	105	Rat	H	E17–E18	0.1	PBS	x	10	37	S-A	P-4762	x	Use	Use
[3]	2017	28	Mouse	TG	6–10 weeks	x	HBSS+	40	30	37	W	x	yes	Use	x
[4]	2009	39	Rat	C	16 weeks	2.0	Hib. A	x	30	30	W	x	x	x	x
[5]	2014	55	Rat	H	E18	3.5	PBS	x	12	32	Wako	x	x	x	Use
[6]	2014	33	Rat	H	E18	x	PDS	20	15–20	37	WBC	PDS	x	Use	Use
[7]	2020	28	Mouse	WB	P0 or P1	N/A	N/A	N/A	10–30	x	BB	N/A	N/A	N/A	N/A
[8]	2017	37	Mouse & Rat	H	P1-3	1.0	L-15	x	x	x	N/A	N/A	x	Use	x
[9]	2014	681	Mouse	H	E16–E17	x	HBSS	20	30–60	37	WB	LS 3126	x	Use	Use
[10]	2017	110	Mouse	C	E14–E15	x	PDS	20	x	x	WBC	x	x	Use	Use
[11]	2015	656	Mouse	C	E17	x	PDS	20	x	x	WB	LK003150	x	Use	Use
[12]	2020	126	Mouse	C, VM, S	E16–E18; P1	x	x	20	20	37	WB	LS003126	x	Use	x
[13]	2018	233	Mouse	C	E16.5	x	PDS	20	x	x	WBC	PDS	x	Use	Use
[14]	2013	166	Mouse	SC	E12.5	x	PDS	20	x	x	W	PDS	x	Use	Use
[15]	2019	101	Mouse	H	P0 or P1	0.5	PGB	x	20	37	x	x	x	x	Use
[16]	2020	136	Mouse	C	E17	x	PDS	20	x	x	WBC	PDS	x	Use	Use
[17]	2012	307	Mouse	C	P0	N/A	x	N/A	x	x	W	x	x	x	x
[18]	2011	117	Mouse	RGC	P7–P8	x	N/A	15	30	37	WB	x	x	Use	Use
[19]	2020	188	Mouse	H	P1	N/A	x	N/A	20	27	x	x	x	x	x
[20]	2021	101	Mouse	Ce	N/A	N/A	x	N/A	30	x	x	x	x	x	x
[21]	2017	180	Mouse	H	E16–17	N/A	HBSS+	N/A	x	x	WB	x	x	x	x
[22]	2020	121	Mouse	H	E16–E18	N/A	x	N/A	45	37	WBC	LS003126	x	Use	x
[23]	2016	167	Rat	H	P0	x	x	x	x	x	x	x	x	x	x
[24]	2014	1275	Mouse	H	P0–1	x	x	50	5	x	WB	x	x	x	x
[25]	2022	103	Rat	C, H, VZ	E18	2.0	x	x	30	37	BB	PAP	x	x	x
[26]	2021	165	Mouse	C and H	E15.5–16.5	N/A	HBSS	N/A	12	37	WBC	LS003127	x	Use	Use
[27]	2019	102	Mouse	C	E18	1.0	x	x	10	37	S-A	76220	x	x	x
[28]	2018	102	Rat	C	E18	x	x	N/A	x	x	WB	LS003126	x	Use	Use
[29]	2016	301	Mouse	H	E18	x	PDS	20	x	x	WB	PDS	x	Use	Use
[30]	2013	161	Mouse	H	P0–P1	x	EBSS	20	30	37	x	x	x	x	x
[31]	2018	344	Mouse	H	P0–P1	x	x	50	6–8	x	WB	x	x	x	x
[32]	2013	485	Mouse	C	E15.5–16.5	x	HBSS	N/A	x	37	W	x	x	x	Use
[33]	2018	363	Rat	H	P0–1	x	x	x	x	x	x	x	x	x	x
[34]	2017	370	Mouse	C	E13.5	N/A	x	N/A	10	37	MB	130-092-628	x	x	x
[35]	2017	212	Mouse	LC	P1	1.0	Custom	x	30	37	S-A	P4762	x	x	Use
[36]	2012	138	Mouse	H	2,4,8,11,21 mo.	2.0	Hib. A	x	30	30	W	x	x	x	x
[37]	2011	109	Mouse	C	1 mo.	x	Custom	x	30	30	x	x	x	x	x
[38]	2017	103	Mouse	DG/H	3–4 mo.	x	EBSS	20	60	37	x	x	x	x	Use
[39]	2014	278	Mouse	H	P0	x	x	x	x	x	x	x	x	x	x
[40]	2016	154	Mouse	C	P0	x	x	100	30	37	x	x	x	x	x
[41]	2018	130	Mouse	H	P0	x	x	20	x	x	S-A	x	x	x	x
[42]	2009	137	Mouse	C	E17–E18	x	x	x	45	37	WB	x	x	x	x
[43]	2010	113	Mouse	C and S	E18	x	PDS	N/A	x	x	W	PDS	x	Use	Use
[44]	2016	443	Mouse	H and C	E16–18	x	x	x	x	x	WBC	x	x	x	x
[45]	2020	127	Mouse	WB	N/A	x	x	20	20	37	x	x	yes	x	x
[46]	2012	129	Mouse	H	P0-P2	N/A	x	N/A	x	x	x	x	x	x	x
[47]	2010	34	Mouse & Rat	RGC	X	x	DMEM	30-35	10	37	W	x	x	Use	x
[48]	2012	79	Rat	C	E18	N/A	DMEM	x	30	37	x	x	x	x	Use
[49]	2017	53	Human	C	N/A	x	x	20	10–15	37	WB	x	x	x	x
[50]	2021	55	Mouse & Rat	C	E18	x	x	x	x	x	x	x	x	x	x

Footnotes: Age = Embryonic (E), Postnatal (P). Brain Region = Cortex (C); Cerebrum (Ce); Dentate Gyrus (DG/H); Hippocampus (H); Locus Coeruleus–Pons (LC); Retinal Ganglion Cells (RGC); Striatum (S); Spinal Cord (SC); Trigeminal Ganglion (TG); Ventral Mesencephalon (VM); Ventricular Zone (VZ); Whole Brain (WB). Media = Hank’s balanced salts solution (HBSS), phosphate-buffered saline (PBS), papain dissociation system (PDS), Earle’s balanced salts solution (EBSS), phosphate-glycine buffer (PGB), Dulbecco’s Modified Eagle Medium (DMEM). Manufacturer = Worthington (W); Worthington Biochem. (WB); Worthington Biochem. Corp. (WBC); Sigma-Aldrich (S-A); BrainBits (BB); Miltenyi Biotec (MB). All = Not reported (X), Not Available (N/A), Use/report use (Use), DNase (Yes) not used (x).

## Data Availability

The raw data supporting the conclusions of this article will be made available by the authors on request.

## Abbreviations

The following abbreviations are used in this manuscript:

ECM	Extracellular matrix
EDTA	Ethylenediaminetetraacetic acid
HEPES	4-(2-hydroxyethyl)-1-piperazineethanesulfonic acid
RNA	Ribonucleic acid
